# Regional Similarities in Seasonal Mortality across the United States: An Examination of 28 Metropolitan Statistical Areas

**DOI:** 10.1371/journal.pone.0063971

**Published:** 2013-05-29

**Authors:** Adam J. Kalkstein

**Affiliations:** Department of Geography and Environmental Engineering, United States Military Academy, West Point, New York, United States of America; University of Utah, United States of America

## Abstract

Human mortality exhibits a strong seasonal pattern with deaths in winter far exceeding those in the summer. While the pattern itself is clear, there have been very few studies examining whether the magnitude or timing of seasonal mortality varies considerably across space. Thus, the goal of this study is to conduct a comprehensive geographic analysis of seasonal mortality across the United States and to uncover systematic regional differences in such mortality. Unique seasonal mortality curves were created for 28 metropolitan statistical areas across the United States, and the amplitude and timing of mortality peaks were determined. The findings here indicate that the seasonality of mortality exhibits strong spatial variation with the largest seasonal mortality amplitudes found in the southwestern United States and the smallest in the North, along with South Florida. In addition, there were strong intra-regional similarities that exist among the examined cities, implying that environmental factors are more important than social factors in determining seasonal mortality response. This work begins to fill a large gap within the scientific literature concerning the geographic variation and underlying causes of seasonal mortality across the United States.

## Introduction

Research has uncovered distinctive temporal patterns in human mortality, with the highest mortality rates in the winter and lowest in the summer [1], [2], [3], [4]. This pattern is consistent across much of the world, including the entire United States. The specific causal mechanisms behind the seasonal mortality cycle are complex and range from high levels of infectious disease [5], [6], to the impacts of snow and cold among the elderly [7], to higher incidence of heart disease in the winter [8], and beyond [9]. Understanding the spatial and temporal variability of seasonal mortality is an important undertaking, as it can assist in improving healthcare and ultimately saving lives.

Originally, it was hypothesized that the winter peak in mortality was due to colder weather which kept people indoors and in close confines. As a result, many believed that infectious diseases such as influenza could be more easily spread among individuals in the wintertime, thus increasing mortality [10], [11]. However, as seasonal mortality was dissected across different geographical regions, it was found that cities with moderate climates have generally similar seasonal patterns in mortality compared to locations with much larger temperature fluctuations throughout the year. In fact, cities such as Phoenix, AZ and Miami, FL, locations in which summer conditions are conducive to remaining indoors (rather than winter), also exhibit a distinctive seasonal mortality trend with heightened mortality in the winter.

Nevertheless, these seasonal trends vary, as do their causal mechanisms, from one region to the next, and more moderate climates within the United States have a slightly more pronounced seasonal cycle in mortality [1]. Clearly, this illustrates that the seasonal mortality cycle is much more complex than originally thought, and its causes are multifaceted.

Considering the uncertainty surrounding seasonal mortality, it is necessary to determine how these variations in seasonal mortality vary by space. Thus, the purpose of this research is to conduct a geographic analysis of seasonal mortality across the United States and to uncover systematic regional differences in such mortality. More specifically, the goals of this study are: (1) To develop average seasonal mortality curves for numerous cities across the United States, focusing on the dates of the annual maxima and minima for each location along with the amplitude of each city’s unique mortality curve. (2) To determine if seasonal mortality varies across space in a systematic way; that is, do geographical regions experience similar timing and magnitude in the seasonal mortality cycle? Some previous studies suggest that more moderate climates often exhibit the largest seasonal amplitude in mortality [1], [12], [13]. However, others refute this notion [14], [15]. It is also likely that variations in the timing of seasonal mortality also exist across the United States, although scant research has been conducted on this topic.

Historically, geographical differences in seasonal mortality have been underemphasized, as research generally concentrates on the impacts of a few extreme mortality events.

## Data and Methodology

**Figure 1:**
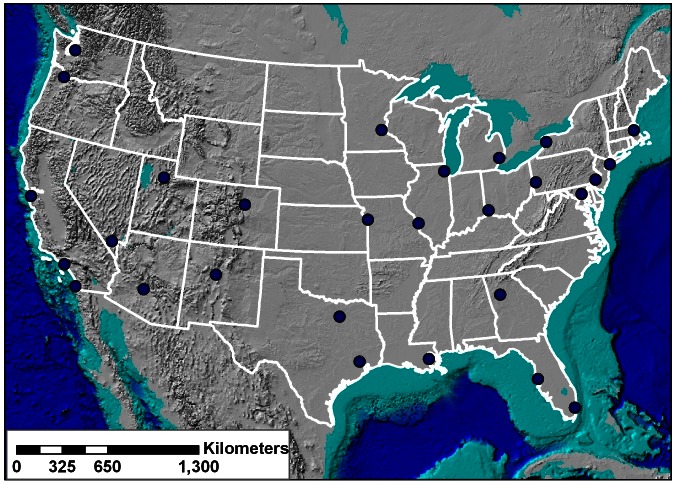
Locations of the 28 MSAs across the United States.

The data necessary for an evaluation of seasonal mortality across the United States were provided by the National Center for Health Statistics (NCHS) and include daily mortality counts that span from 1975 through 2004, the most recent data available at the time of this study. These daily mortality data are grouped by metropolitan statistical area (MSA), as defined by the 2000 population census. Each MSA includes the county or counties that comprise each city and the surrounding area, and 28 MSAs are examined in this study, enough to provide a detailed geographical analysis of seasonal mortality across the United States (Table 1, Fig. 1). These specific cities were selected based upon data completeness and geographic location to permit an evaluation of spatial differences that may exist in seasonal mortality. Additionally, nearest neighbor analysis was conducted to ensure that cities were not geographically clustered in any one location. The resulting nearest neighbor statistic is 1.31, indicating that the selected cities are randomly to uniformly distributed across the United States. Mortality data were incomplete in Dallas for 1989, 1990, and 1991 and for 1990 and 1991 in Houston, and these years were not examined for these two cities.

**Table 1:** List of the 28 Metropolitan Statistical Areas (MSAs).

Albuquerque, NM	Miami, FL
Atlanta, GA	Minneapolis, MN
Baltimore, MD	New Orleans, LA
Boston, MA	New York City, NY
Buffalo, NY	Philadelphia, PA
Chicago, IL	Phoenix, AZ
Cincinnati, OH	Pittsburgh, PA
Dallas, TX	Portland, OR
Denver, CO	Seattle, WA
Detroit, MI	Salt Lake City, UT
Houston, TX	San Diego, CA
Kansas City, MO	San Francisco, CA
Las Vegas, NV	St. Louis, MO
Los Angeles, CA	Tampa, FL

### Age-adjusted Standardization

It is necessary to standardize the raw mortality data to permit a proper spatial evaluation across geographic domain. Raw mortality totals are highly dependent on the age structure of a given location, and an MSA with an older population will have consistently higher death rates regardless of other factors. Thus, direct comparisons of raw mortality totals cannot be made between cities with varying age structures, or within a city over a long period of time.

This study utilizes a direct standardization method, and the mortality rates are standardized to the population distribution of the United States in 2000 across ten distinct age segments (<5, 5–14, 15–24, 25–34, 35–44, 45–54, 55–64, 65–74, 75–84, >84), as discussed in Anderson and Rosenberg [16]. To adjust for population changes within each MSA throughout the period of record, population totals for each age group were compiled from the United States Census for 1970, 1980, 1990, and 2000. A linear interpolation was then used to estimate population counts for the intervening years, a methodology consistent with previous studies [1]. The final age-adjusted death rate (ADR) is presented as daily deaths per standard million.

Throughout the period of record, there were instances in which disasters unrelated to seasonal mortality (e.g. plane crash) had a profound impact on the daily mortality counts, and these outliers were removed. In all, mortality data were discarded for only 25 days covering 17 cities. These outliers were identified as having a daily adjusted mortality total for injury and poisoning deaths with a z-score of over 10. Only injury and poisoning deaths were used to determine outliers since they were the best indicator of a disaster unrelated to seasonal mortality. Each of the outliers removed had a known source, with the most common being airline crashes. Other days removed resulted from building collapses, boat accidents, mass murder and suicide, natural disasters (non weather-related), and terrorist attacks.

### Creation of Seasonal Mortality Distributions

A key aspect of this study is the creation of seasonal mortality “curves” for each MSA. First, using daily ADR, average daily mortality rates across the period of record were calculated and plotted for each MSA. For example, for 1 January, assuming 30 years of mortality data, the mean for the 30 daily 1 January death rates was calculated. This was repeated for every day of the year, creating 365 daily mortality counts in each MSA. These data were then smoothed using a 15-day running mean. 15 days were chosen to balance the conflicting needs of (a) smoothing fluctuations due to weather and other factors and (b) comparing mortality only within similar times of the year. On the one hand, averaging over as long a period as possible creates a smoother, and likely more accurate mortality curve. Conversely, averaging over too long a period could lead to including days that are different than the day in question due to seasonal differences in mortality. 15 terms, or one week on either side, represents a compromise between these two concerns. Additionally, sensitivity analysis was conducted to confirm that mortality curves created using a 15-day running mean did not differ substantially from those created using an 11 or 19-day smoothing procedure.

In all, 29 mortality curves were created, one for the United States and each MSA, and these curves represent an accurate depiction of the general shape of a given city’s seasonal mortality response. Additionally, the seasonal mortality amplitude, the percentage difference between the winter peak and the summer valley, was calculated. However, it was found that amplitudes calculated using this methodology consistently underestimated the actual amplitudes observed on a year-to-year basis. This was a result of small annual fluctuations in the timing of the winter peak, which when averaged together to create the overall mortality curve, resulted in a lower, plateau-shaped winter peak rather than a more abrupt feature that consistently occurred from year-to-year.

Partially to rectify this situation, it is also necessary to create an average mortality curve for each year in each location. This allows for a temporal analysis in seasonal mortality across the period of record, as well as a more accurate way to calculate both the average timing and amplitude in mortality for a given city. There have been considerable changes in mortality from the mid-1970’s through 2004, and considering that these curves are created for each year over the period of record, it is easier to control for any fluctuations in mortality over time. Previous studies have controlled for these changes using a linear adjustment, but many changes in mortality over time do not follow a linear trend, and this methodology was rejected. Rather, age-adjusted mortality was compared to that category’s average mortality count for each given year. To provide an example, assume that in a given city in 1980, there were on average 20 deaths per standard million per day. If on 4 July 1980 there were 25 age-adjusted deaths, this date would be recorded as 0.25, or 25 percent above average in that year. This methodology was conducted for total ADR, thus controlling for temporal changes in mortality across the period of record for each MSA.

The daily percentages calculated for total ADR, as described above, were then used to create mortality curves for each city for each year. A 29-day running mean, or two weeks on each side of the day in question, was used to smooth these data. 29 days were chosen, rather than the 15 days used for the overall mortality curves created for each city, as a result of a significantly smaller sample size. In this case, only one year at a time was being examined, as opposed to all years for the mortality curves created earlier.

The final complicating factor involved in creating annual mortality curves for each city is that most mortality peaks occur around the New Year. Thus, using the methodology described above, if a mortality peak occurred on 31 December 1979, it would often count as the highest mortality total for 1979, as opposed to 1980. Additionally, mortality counts on 31 December 1979 would be compared to 1979 averages while 1 January 1980 would be compared to 1980 averages, thus creating small but abrupt changes. To solve these two problems, these mortality curves were created from 1 November through 31 October. Further, the daily percentages for ADR, which were used for the annual mortality curves for each city, were also calculated by comparing the daily values to the average annual values from 1 November through 31 October. Thus, using an example described earlier, assume a given city had on average 20 deaths per standard million per day from 1 November 1979 through 31 October 1980. If on 4 July 1980 there were 25 age-adjusted deaths, this date would be recorded as 0.25, or 25 percent above average in that year. Mortality curves for each year in each city can now be created, although there were no curves created for 1975 since the first day in the period of record is 1 January 1975, and a mortality curve for 1975 would require data beginning on 1 November 1974. To summarize, these curves are controlled for temporal changes in mortality, are calculated from daily ADR percentages, and are smoothed using a 29-day running mean. A unique curve was created for each year and for each city spanning from 1976 through 2004.

### Calculation of Amplitude and Timing

The annual creation of mortality curves for each MSA allows for the calculation of year-to-year amplitude in this curve, along with the timing of the winter peak in mortality. The amplitude for each year for each city was calculated by finding the difference between the maximum daily value for a given year and the minimum daily value. For example, if the highest daily mortality total for a given year was 25 percent above average while the minimum summer low was 20 percent below average, the amplitude for that year would be recorded as 45 percent. Average amplitude for each MSA was calculated by finding the mean of all 29 annual amplitudes calculated across the period of record. Median was also examined and found to be nearly identical.

The timing of the date of maximum mortality for each city was found by recording the date at which the daily maximum mortality value occurred in a given year on the annual mortality curve. The average date of maximum mortality in each city was calculated by taking the median of each year’s value across the period of record. Although the mean was also examined, median was found to be a more accurate representation of the actual timing of maximum mortality since these values varied substantially from year-to-year. Thus, while one or two unusual dates could have a profound impact on the mean, they had little impact on the median. These methods were repeated to find the dates of minimum mortality as well.

### Regionality

An important aspect of this study is to determine how seasonal mortality patterns vary geographically across the United States. The first goal is to determine if there is regionality to the seasonal mortality curves for each MSA; in other words, do cities located near each other experience similar annual cycles in mortality? To characterize and compare mortality curves for each city, three variables were examined: average amplitude, the average date of maximum mortality, and the average date of minimum mortality. These three variables were each plotted across the United States, and maps were created highlighting spatial variation. The standard deviations were also plotted for each of these three variables to help determine if year-to-year patterns in mortality are more variable in certain parts of the country.

An additional regional analysis was conducted to determine how geographic distance between cities impacts year-to-year variation in seasonal mortality. Do neighboring cities experience similar cycles in mortality on an annual basis? To examine if year-to-year similarities in seasonal mortality exist among various cities, annual mortality amplitude and timing were compared among MSA’s. First, scatterplots were created for both amplitude and the date of maximum mortality for every possible combination of two cities. For example, a scatterplot was created comparing annual amplitudes between Albuquerque and Atlanta, then Albuquerque and Baltimore, then Albuquerque and Boston, until every possible combination of cities was plotted. In all, there were 378 combinations (27+26+25+ …. +3+2+1). For each scatterplot, linear correlation was used to record the r-squared value; a high r- squared for amplitude, for example, would indicate that that a high mortality amplitude in a given city in one year would likely correspond to a high amplitude in that same year for another city. This methodology was then repeated for the date of maximum mortality to determine if there were any year-to-year relationships between cities in terms of when the winter mortality peak occurred. Once the r-squared values were calculated for all 378 city combinations for both amplitude and timing, the geographic distance between each city was computed. Finally, geographic distance was then compared to the mortality amplitude and timing r-squared values. It is expected that a negative relationship will result, suggesting that the closer each city is to another, the stronger the relationship will be between their year-to-year mortality cycles.

## Results

### Mortality Curves

**Figure 2:**
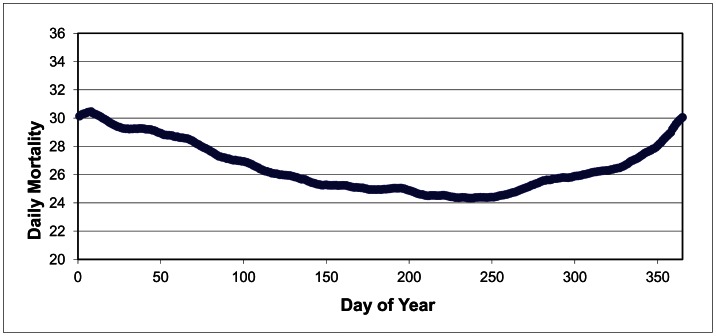
Average daily mortality across the United States, in deaths per standard million, from 1 January (1) to 31 December (365).

Each of the 28 cities examined experiences a strong seasonal trend in mortality, with higher mortality in the winter and lower in the summer. For the United States as a whole, mortality peaks on 8 January at 30.46 deaths per standard million and reaches its summer low on 25 August at 24.34 deaths (Fig. 2). Additionally, the slope of the mortality curve is rather gradual, particularly the observed decrease in mortality from mid-January throughout the summer. There is evidence of a two-step increase in wintertime mortality, and this feature is also observed in many of the cities examined. Mortality begins to increase steadily in early September, flattens out from mid-October through mid-November, and then increases rapidly until the first week of January.

**Figure 3:**
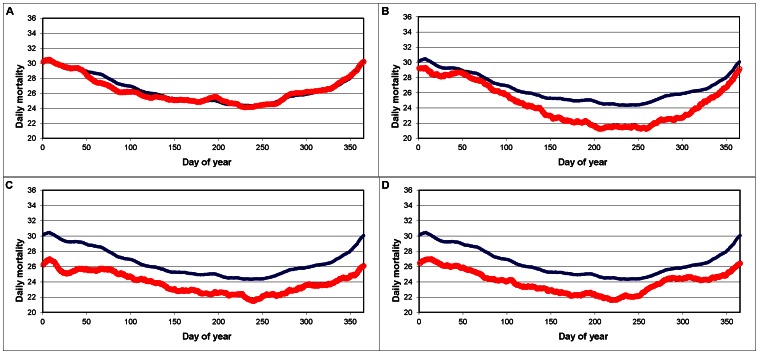
Average daily mortality (red) for New York City (A), Phoenix (B), Seattle (C), and Minneapolis (D) compared to the United States (blue), in deaths per standard million, from 1 January (1) to 31 December (365).

While the general winter-high/summer-low shape of the seasonal mortality curves was noted for each MSA, there were some substantial differences. For example, cities prone to heat-related illness, such as Chicago, New York, and Philadelphia, experience a small summer peak as a result of increases in heat-related mortality. Other cities, such as Phoenix and Los Angeles, experience particularly rapid increases in mortality throughout the fall, while this rise is more gradual in other locales such as Seattle and Portland. Some cities exhibit an interesting two-step rise in mortality throughout the fall, and this is evident in the mortality curves of Minneapolis and Detroit. Finally, while most cities experience a gradual decline in mortality from mid-January through the summer, the seasonal changes in mortality are much more abrupt in several cities such as Miami. Here, mortality is high in the winter and low in the summer with very little transition between the two (Fig. 3).

An important aspect of this study is to examine the amplitude of seasonal mortality, or the percentage difference between the winter maximum and summer minimum. However, as mentioned previously, simply calculating the amplitude of each city’s mortality curve consistently underestimates the actual seasonal mortality amplitude. For example, the amplitude of the mortality curve for the entire United States averaged over the period of record in Fig. 2 is just over 23 percent. However, averaging each individual year’s amplitude in mortality produces a higher and more accurate result; using this methodology, the average amplitude across the United States is 31.7 percent (Table 2). Not surprisingly, the average annual amplitude in seasonal mortality varies substantially from one city to the next, ranging from 42.1 percent in Albuquerque, 40.3 percent in Las Vegas, and 40.1 percent in Phoenix to 26.0 percent, 27.3 percent, 27.8 percent, and 28.3 percent in Chicago, Miami, Detroit, and Minneapolis, respectively (note the general homogeneity in regionality). The median was also calculated for amplitude, and this analysis yielded similar results.

**Table 2:** Average annual amplitude and the average dates of maximum and minimum mortality.

Location	Average Annual Amplitude(Mean; In Percent)	Average Date of MaximumMortality (Median)	Average Date of MinimumMortality (Median)
United States	31.7	1/15	8/13
Albuquerque	42.1	1/21	8/5
Atlanta	32.9	1/14	8/6
Baltimore	30.6	1/16	8/18
Boston	31.2	1/14	8/16
Buffalo	31.8	1/15	8/10
Chicago	26.0	1/10	7/24
Cincinnati	33.0	1/8	8/10
Dallas	31.0	1/27	8/24
Denver	34.4	1/14	8/14
Detroit	27.8	1/12	8/14
Houston	28.9	1/14	8/25
Kansas City	30.6	1/28	8/2
Las Vegas	40.3	1/11	8/12
Los Angeles	31.3	1/11	8/17
Miami	27.3	2/6	9/4
Minneapolis	28.3	1/21	7/31
New Orleans	30.9	1/13	8/7
New York City	28.7	1/12	8/18
Philadelphia	30.3	1/15	8/20
Phoenix	40.1	1/19	8/17
Pittsburgh	29.7	1/23	8/13
Portland	28.9	1/8	8/27
Salt Lake City	33.0	1/12	8/19
San Diego	32.3	1/9	8/7
San Francisco	31.9	1/11	8/25
Seattle	28.8	2/3	8/25
St. Louis	30.9	1/10	8/8
Tampa	34.5	1/24	8/25

The specific timing of seasonal mortality across the United States was also examined. Similar to the amplitude, the dates of maximum and minimum mortality were calculated by averaging these dates for each individual year. However, unlike amplitude, in this case it was advantageous to use the median, as discussed earlier. Using medians to calculate the timing in seasonal mortality, the average date of maximum mortality for the United States was 15 January, while the summer mortality minimum occurred on 13 August (Table 2). Note that there were small differences in the timing of seasonal mortality based upon the methodology employed; averaging mortality across the entire period of record resulted in an observed peak for the United States of 8 January. On the other hand, first calculating the timing during each, individual year and then finding the median of these values produced a date of 15 January. The latter method, thought to be more indicative of actual annual mortality responses, is used throughout the remainder of the study.

Much like the seasonal mortality amplitudes, the timing of seasonal mortality varied considerably from one city to the next (Table 2). The cities with the earliest peaks in winter mortality were Portland, Cincinnati, and San Diego with their peaks occurring on January 8, 8, and 9, respectively. Seattle and Miami had the latest winter mortality highs, which occurred on 3 February and 6 February.

**Figure 4:**
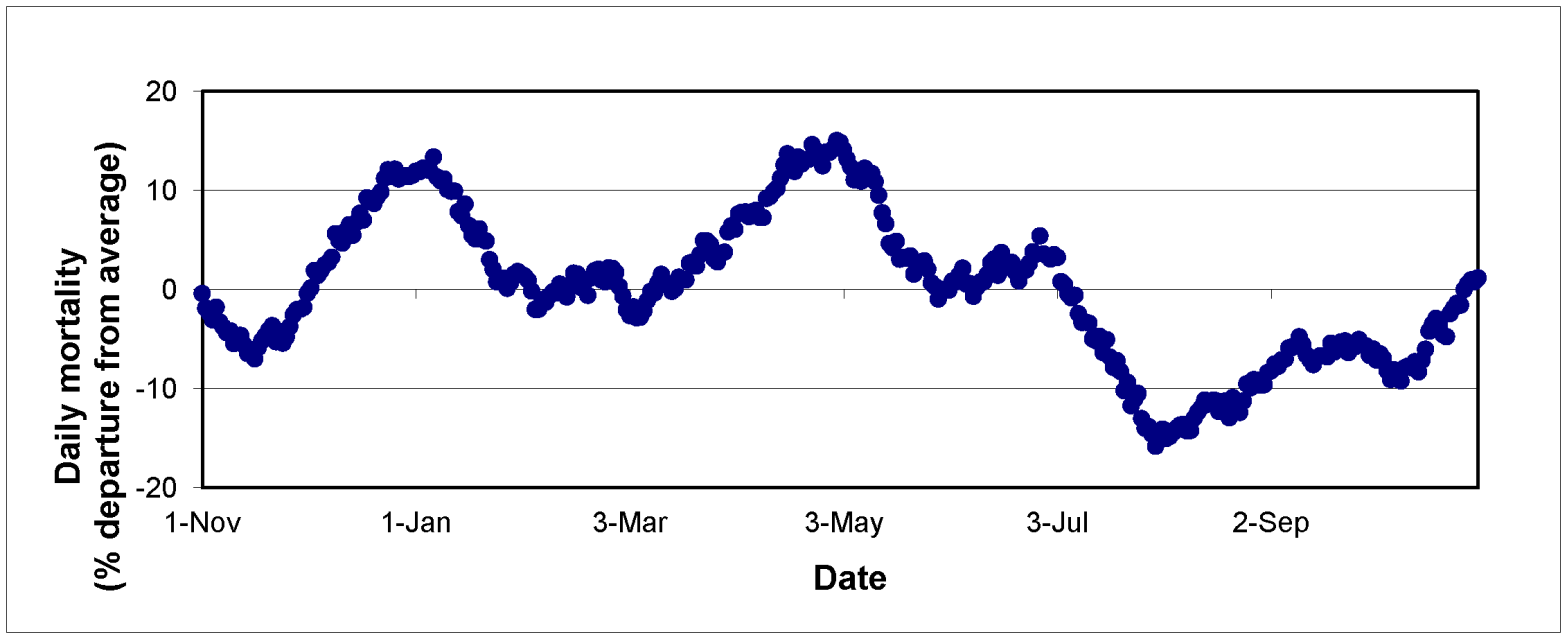
Daily mortality in Seattle from 1 November, 1981 through 31 October, 1982, in percentage departure from average.

An important complicating factor in analyzing the timing of the winter peak in seasonal mortality is an observed dual peak that occurred occasionally throughout the period of record. For example, Fig. 4, which spans from 1 November 1981 through 31 October 1982, demonstrates this phenomenon in Seattle during the winter and spring of 1982. While this unusual event has little effect on the median dates of the winter peaks listed above, it plays a much more important role in any regional or temporal analysis of seasonal mortality timing. To provide an example, although the 1982 mortality curve in neighboring Portland looks nearly identical to that in Seattle, the winter maximum occurs during the first peak several months earlier. Similarly, as noted above, the average date of maximum mortality occurs about a month later in Seattle compared to Portland, a direct result of dual mortality peaks. This phenomenon will be discussed later in greater detail, but it is important to note that while the dates of maximum mortality between two cities might appear very different in a given year, their actual mortality curves could be highly similar. Additionally, it is likely that these unusual curves resulted in weaker results when examining the relationship between geographical distances among MSAs to the year-to-year timing of the winter mortality peak, as discussed below.

### Regionality

**Figure 5:**
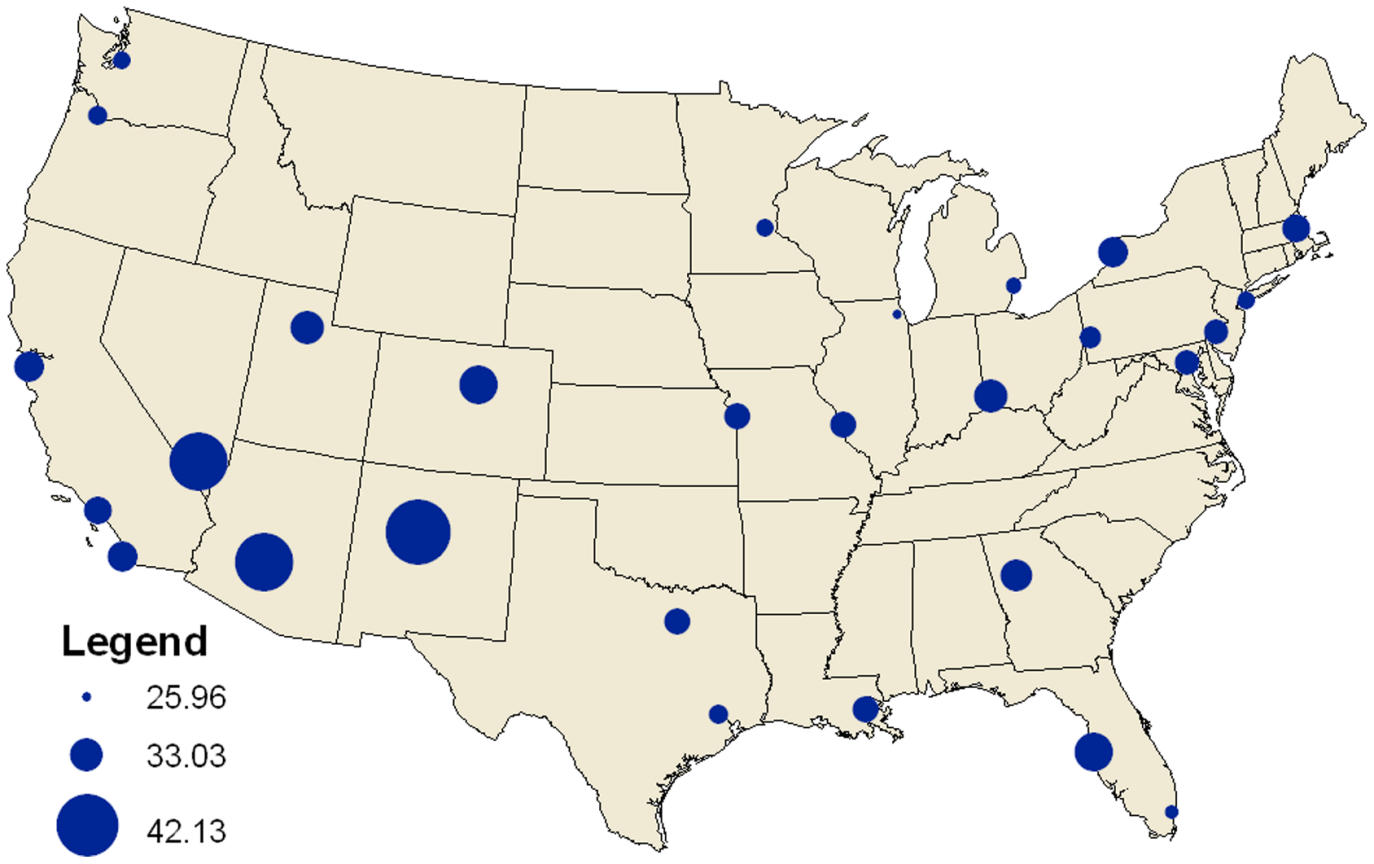
Average mortality amplitude across the United States.

The final goal in this study is to determine how seasonal mortality varies geographically across the United States and to examine if any regionality exists. First, average amplitude in seasonal mortality was calculated for each MSA, and then it was plotted throughout the United States, thus highlighting any geographical variation (Fig. 5). Amplitude exhibits a strong spatial signal with much higher values in the Southwest and the smallest in extreme south Florida as well as the North. Additionally, the standard deviation of year-to-year amplitude is also highest in the Southwest, implying larger annual fluctuations in this region. The average date of maximum mortality was plotted for each city, but unlike mortality amplitude, very little regionality exists, with the exception being later dates in South Florida.

A final spatial analysis was conducted to determine if geographical distance between cities influences year-to-year patterns in seasonal mortality. This was accomplished by first calculating the r-squared value using linear regression for year-to-year amplitude between two cities and then repeating this methodology for each corresponding combination of two cities. In all, there were 378 combinations of city pairs. A high r-squared value indicates that there is a strong relationship between amplitude from year-to-year between cities, or in other words, a high amplitude in one city in a given year would likely mean that a high amplitude existed in the next city during the same year. A low r-squared would indicate the opposite, suggesting that there is little relationship among cities in annual amplitude. All 378 r-squared values were then compared with each city combination’s geographic distance from each other. This methodology was then repeated for the annual timing of seasonal mortality.

**Figure 6:**
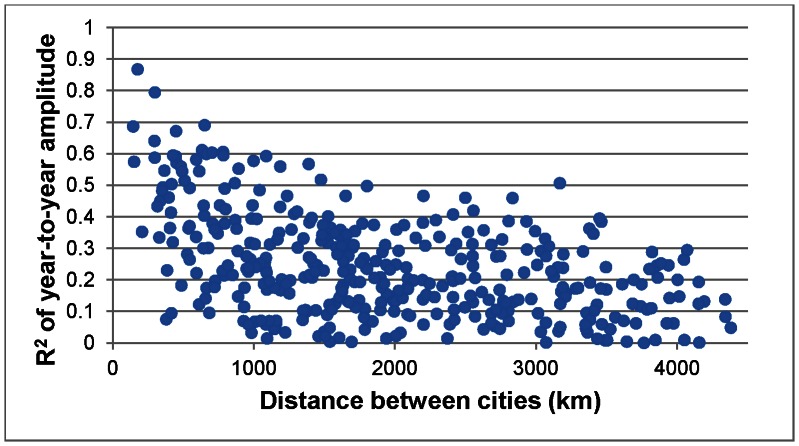
Relationship between year-to-year amplitude and geographic distance among cities. Higher r-squared values indicate larger relationships in amplitude.

**Figure 7:**
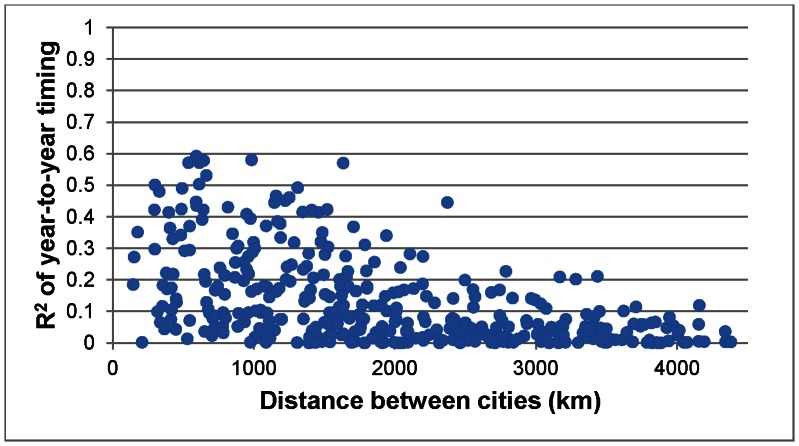
Relationship between year-to-year timing of the winter mortality peak and geographic distance among cities. Higher r-squared values indicate larger relationships in timing.

There is a strong relationship between geographic distance and annual amplitude among cities, indicating that cities located in close proximity to one another experience similar mortality amplitudes from year-to-year (Fig. 6). This relationship is highly statistically significant (*p* = 0.000) with an r-squared of 0.219. It appears as if this relationship is not completely linear, with both logarithmic (r-squared = 0.294) and third-order polynomial (r-squared = 0.308) trends providing better fits. Interestingly, the relationship between distance and the annual timing of the mortality peak is even more striking, indicating that cities located near each other experience similar dates of maximum mortality (Fig. 7). This relationship is also highly significant (*p* = 0.000) with an r-squared value of 0.265. The strong relationship in year-to-year timing of the seasonal mortality peak is particularly surprising given that the “dual peak” signature discussed earlier would weaken these results considerably. Clearly, there is a strong regional homogeneity in amplitude and timing within the cities’ annual mortality curves.

## Analysis and Discussion

The major underlying finding of this evaluation is that there is a strong seasonal pattern to mortality in the United States that is generally regionally homogenous but varies across space. This study confirms that overall seasonal mortality patterns across the country are similar to those found elsewhere in the Northern Hemisphere including both Europe and Asia [2], [4], [17]. Although similar, it is still quite striking that average mortality values in the winter can exceed those in the summer by up to 25 percent in some locales, with the largest values found in the Southwest. Taken as a whole, this strong seasonal pattern indicates that the mechanisms responsible for increases in winter mortality dwarf the impact of heat on summer mortality, although individual heat waves still account for substantial short-term mortality spikes.

### Seasonal Mortality Curves

The specific timing of the United States mortality curve seems to refute several studies that suggest a rapid increase in mortality occurs during the holiday season [18], [19]. In this analysis, the average nationwide date of maximum mortality is 15 January, nearly three weeks after Christmas, and even later in many cities.

Not surprisingly, the mortality curves created for each MSA vary considerably from one city to the next, and many interesting features not found in the United States curve become evident. Examples of this include a two-step winter rise in mortality found in cities such as Minneapolis and Detroit, a particularly steep winter rise in places like Los Angeles and Phoenix, a dual-peak curve found in Seattle and San Francisco, and an unusual plateau-shaped curve found in Miami. Each city’s unique shape is likely caused by a combination of climate, infrastructure, and social factors, and a major goal of this research is to help determine the role that each of these variables play.

First, the two-step winter rise in mortality is generally a northern-city phenomenon. While this feature is also found in numerous other locations, and to a lesser extent in the United States curve, it is much more pronounced for cities in the North. In these instances, a sharp mortality rise occurs during the first several weeks of September, followed by little change in mortality over the next month or two. Previous research linking weather to human-health responses has consistently found that the first extreme weather day of a season often has a profound physiological response. For example, heat-related mortality is most pronounced during the first heat wave of the year, when the body is still unacclimatized to extreme heat [20]. Similarly, hospital admissions of asthma increase dramatically during the first cold spell of the year [21]. It is possible that the sudden, early-September increase in winter mortality found in these northern cities is due to the season’s first cold weather, which occurs when the body is still adjusted to the summer warmth. The cold would be a relative shock to many, resulting in increased mortality among susceptible portions of the population. Additionally, the relatively flat period following the initial increase in mortality might be related to a similar phenomenon observed following heat waves. In these cases, mortality often drops significantly following a heat wave, as the heat wave proved to be fatal to a portion of the susceptible population who would have passed away during the upcoming weeks, regardless of the weather [22]. This effect is referred to as mortality displacement, and it is possible that a similar, wintertime event is being observed for cities in the North. The subsequent rise in mortality, after the plateau, can be attributed to the more typical winter increase in mortality found across all remaining cities. Since the comments above are based on circumstantial evidence, it is clear that this phenomenon needs to be evaluated further.

Unlike the northern cities, a particularly steep slope (without any plateau) leads up to the winter mortality peak in nearly all southwestern cities including Phoenix, Los Angeles, and Las Vegas. Additionally, the winter rise commences several weeks later in the warmer, southwestern cities, leading to the observed steepness in the slope. The rise in the northern cities begins on approximately 7 September while the rise in Los Angeles commences on approximately 2 October. It appears that the autumn rise in mortality can be partially attributed to the onset of the first cool weather event (for example, Miami has the latest fall rise). In addition, it is possible that the influx of seasonal residents, a phenomenon that will later be discussed in greater detail, might be partially responsible, but it is unlikely to be the sole driving factor behind this abrupt rise. For example, this feature is quite prominent in Los Angeles, a city with relatively few “snowbirds”. Thus, a combination of physical and social factors seems to be playing a role here.

A more complicated phenomenon is the dual winter mortality peak observed during many years in cities such as Seattle. Interestingly, this feature disappears southward along the West Coast. While the dual peak is only somewhat prominent in the average mortality curves, it was particularly strong during several years of this study. A cursory examination of the meteorological conditions present during years in which this dual peak was evident did not provide any insight, and it remains unclear what might be causing this unusual mortality response. Future work is needed to determine why mortality exhibits such atypical winter patterns during certain years in the Pacific Northwest.

Additionally, these dual peaks substantially weakened the results of the analysis examining the timing of the winter peak. For example, when comparing annual mortality curves across the period of record for both Portland and Seattle, they usually looked quite similar. However, when plotting the annual dates of maximum mortality between cities, the r-squared value is 0.002, mainly because the winter peaks occurred during the first apex in one city, but during the second in the other. This, in turn, resulted in no statistical relationship between these two cities from year-to-year. However, the removal of seven years of data in which dual mortality peaks are evident changed the relationship between Seattle and Portland dramatically. In fact, with these years removed, the r-squared value when comparing dates of maximum mortality rose from 0.002 to 0.732, with the trend being highly significant (*p* = 0.000).

Perhaps the most unique mortality curve of all the cities was that of Miami, which had an extended but flat winter mortality high followed by a similarly long and flat summer low with only short, abrupt changes in between. Additionally, both the amplitude and the timing of the mortality peak were unusual with Miami recording the second smallest amplitude, trailing only Chicago, and the latest date of maximum mortality (6 February). It is likely that two important factors are largely responsible for the unusual seasonal mortality response found in Miami: (1) the influx of seasonal winter residents and (2) a weak seasonal mortality response similar to those found in other tropical locales [3], [23].

While this study controls for inter-annual changes in population, it does not adjust for intra-annual changes. Intra-annual changes in population have been a complicating factor in other weather-health relationships. For example, studies examining the impact of heat on human health in Rome, Italy, noted a sharp decline in summer mortality across the city that was attributed to numerous residents leaving on summer vacation [24], [25]. It is possible that a similar event is occurring for southern cities such as Miami, Tampa, Phoenix, and San Diego, all of which experience a large influx of seasonal winter residents. However, estimates of each city’s seasonal population changes vary significantly [26]. Additionally, even if precise population estimates were available, they are not stratified by age, making an age-adjustment procedure impossible. Thus, it is difficult to determine what portion of these cities’ mortality curves is due to an actual seasonal mortality response as opposed to intra-annual changes in population.

Despite the fact that an influx of winter residents likely increases the seasonality of mortality substantially, Miami has the second lowest average amplitude value among all cities. However, other cities with increases in winter population such as Phoenix, Las Vegas, and Tampa all exhibit among the largest amplitudes. This, in addition to the fact that Miami has the latest date of maximum mortality, indicates that Miami has a seasonal mortality response unlike any other in the conterminous United States. Previous research suggests that winter-high/summer-low mortality patterns begin to break down in the tropics, with equatorial locations having small mortality cycles largely due to the timing of the rainy season [3], [23]. Thus, it is hypothesized that the relative lack in seasonal mortality observed in Miami is a result of its tropical climate and location, and not social or other factors unique to the city. Further research needs to be conducted to determine what specifically reduces the seasonality of mortality in Miami, and possibilities include: warm overall climate, little seasonal fluctuation in temperature, lack of freezes, higher solar angle, increased outdoor activity in the winter, and heightened air circulation in homes.

Interestingly, many of these possibilities can be ruled out by comparing Miami’s mortality response with that of Tampa, located only 340 km to the northwest. Tampa exhibits very few of Miami’s unusual mortality characteristics with a high mortality amplitude and near-average date of maximum mortality. Thus, whatever phenomenon is responsible for Miami’s unique curve is not found only 340 km further to the north. It should be noted that Tampa’s winter climate is considerably less tropical than Miami’s, with significantly longer and more intense cold spells and more precipitation. Recent research suggests that decreases in absolute humidity are correlated with the timing and severity of influenza outbreaks [6], [27]. Further, research on guinea pigs demonstrates that influenza transmission is highly affected by changes in both temperature and humidity, with cooler, drier conditions exacerbating the spread of the disease [28], [29], [30], [31]. Despite these findings, however, a more gradual change between these two cities would still be expected. Instead, Tampa’s seasonal mortality response is much more similar to cities much further north. Additional research in this area could potentially lead to important breakthroughs concerning the specific causes of respiratory disease and the spread of influenza in tropical versus mid-latitude locations, a topic that has long eluded scientists.

### Regionality

The regionality of mortality across the United States exhibits strong spatial patterns, generally with the largest seasonality in the Southwest and lowest in the north-central U.S., along with extreme south Florida. A similar pattern also exists with annual mortality amplitude and standard deviation. While there is little doubt that an increase of seasonal residents into the Southwest contributes to the strong homogeneity found among cities in this region, it is not believed to be the only cause. Phoenix, Las Vegas, San Diego, and Albuquerque all exhibit large mortality amplitudes. However, a very important difference between these four cities is that while Phoenix, Las Vegas, and San Diego all likely experience an increase in winter population, Albuquerque does not (Albuquerque sits over 1500 m in elevation resulting in much cooler winters). This is a vital distinction, considering that Albuquerque displays all the unusual seasonality responses found in the Southwest without a substantial influx of winter residents. Thus, it can be assumed that something unusual, beyond the migration of snowbirds, is occurring in the southwestern United States, making it more susceptible to enhanced winter mortality levels.

Several studies provide circumstantial evidence that dust might be partially responsible for the elevated seasonality observed across the Southwest. First, McCarthy [32] suggests that pathogens can survive on dust for extended periods of time, possibly even enduring a week-long transit across the Atlantic Ocean following a Saharan dust storm. Similarly, research on avian influenza notes that dust is a very effective medium for transmission of this disease [33]. Most interesting, however, is a study by Liu et al. [34] which examines the spread of avian influenza in China and parts of Europe. They note that this strain of influenza requires both cool temperatures and high concentrations of dust to be most effectively transmitted. In fact, they conclude that frontal passages and corresponding dust storms were responsible for two severe outbreaks of avian flu in 2005 and 2006. While there are notable differences between the transmission of avian flu and other, more common strains that predominantly impact humans, the lower temperatures and dust found in the Southwest during winter provide an interesting hypothesis to explain the increased seasonality in respiratory disease found in this region. This theory is further supported by the absolute humidity, temperature, and influenza relationships discussed earlier [6], [27], [28], [29], [30], [31]. It is important to note that the climatic conditions across the southwest United States are not completely uniform, primarily due to temperature variations as a result of altitude. However, altitude has less impact on humidity, dust, and frontal passages, all of which have been linked to the spread of infectious disease.

The low amplitude in mortality in the north-central United States also needs further explanation. Although there are small increases in summer heat-related mortality in this region (which reduces amplitude only slightly), the most important contributory factor is the smaller magnitude of the winter peak. With the exception of influenza outbreak years, it appears that the general “hardiness” of northerners is attributed to the lesser sensitivity to the cold weather. Thus, the major amplitude difference between the north-central and southwestern cities is attributed to the higher cold season mortality death rates in the latter. This is an intuitive suggestion, and consistent with why summer mortality is higher in northerly areas where the population is less adapted [35].

It is also plausible that population differences in the north-central United States might also be partially responsible for lower levels of winter mortality. For example, the geographic origin in this region is predominately Western European, with large numbers of people from German, Norwegian, Irish, Polish, and Swedish ancestry [36]. There is some evidence that “cold climate genes” have allowed an adaptation to extreme climatic conditions among certain populations [37], [38] while cultural and technological adaptations to cold climates have been well-documented for years [39].

One final observation that is noteworthy relates to the distance decay in the year-to-year mortality correlations. As described earlier, geographic distance is closely related to annual fluctuations in seasonal mortality, with cities located in close proximity having strong year-to-year correlations. It is strongly suggested that environmental, rather than social factors, are responsible for this. A 2007 study by Borden et al. [40] provides additional evidence for this conclusion. Their research highlighted a variety of social and infrastructure variables in urban areas across the United States that are associated with increased risk of natural disasters. Some of the variables examined included poverty, ethnicity, population growth, gender, urban density, and building age. Although there was some regional homogeneity associated with natural disaster risk, it did not correspond well with the regional similarities observed among human mortality. For example, there were large social differences observed between New York and Boston, Los Angeles and San Francisco, and many other neighboring cities that displayed similar trends in seasonal mortality. In addition, while social and built environment vulnerability was highly similar for Tampa and Miami, their mortality responses varied wildly. Clearly, the strong regionality observed in seasonal mortality cannot be solely attributed to social and/or infrastructural factors.

### Conclusions

The purpose of this study was to conduct a comprehensive geographic analysis of seasonal mortality across the United States. More specifically, the goals of this research were to develop average seasonal mortality curves for numerous cities across the United States and to determine if the seasonal mortality curves vary across space in a systematic way.

First, mortality data from 28 metropolitan statistical areas (MSAs) across the conterminous United States were age-standardized to adjust for varying age structures found between cities. Average seasonal mortality curves were then produced for each MSA to better understand each city’s unique seasonal mortality response. After adjusting for changes in death rates over time, mortality amplitude, the maximum mortality in a given year minus the minimum, was calculated for each year, and the dates of maximum and minimum mortality were also recorded.

Regionality was also considered, and maps were created for the United States, demonstrating geographical variations in seasonal mortality. Further, geographic distance between cities was examined to determine if space influences year-to-year relationships in mortality timing and amplitude between cities.

The major findings of this study are as follows:

- This study confirms that the entire United States experiences heightened mortality in the winter and lower in the summer, although the differences between the two seasons vary considerably on a regional basis.
- The largest seasonality in mortality is found in the southwest United States, a phenomenon not solely attributed to an influx of winter residents, and very likely caused in part by increased transmission rates of influenza as a result of cool, dry, and dusty conditions.
- The smallest seasonality in mortality is experienced across the North, as well as extreme South Florida.
- A more “tropical” pattern in mortality has been uncovered; Miami is unique among U.S. cities in its seasonal mortality response. It appears as if patterns in seasonal mortality begin to break down at this latitude, similar to those found in other tropical cities around the world.
- Many northern cities experience a two-step rise in winter mortality, possibly caused by the initial onset of cold winter weather.
- Geographic distance is closely related to annual fluctuations in seasonal mortality, with cities located in close proximity having strong year-to-year correlations.

The overall strength of the intra-regional similarities was stronger than expected, and this suggests that environmental factors, rather than social, play the most important role in seasonal mortality variation. Another surprise was the relative isolation of Miami, which exhibited a more “tropical” mortality response. This can be a vital tool in determining what environmental factors affect influenza transmission since Tampa, located only 340 km to the north, displays very different characteristics.

The largest limitation of this study was separating intra-annual population changes from actual seasonal mortality responses. Although the effect of “snowbirds” was hypothesized, exact age-stratified counts, necessary for a more exact calculation, were impossible to obtain.

This research starts to fill a large gap within the scientific literature, and the findings are highly relevant to those who seek a better comprehension of seasonal mortality variability across the United States. For example, health practitioners across the country now have more accurate information concerning when mortality will increase and how high the increase will be. Medical officials in the Southwest need to be aware that this region is at elevated risk to strong winter increases in mortality, and there is increasing evidence that the dust and cool, dry weather found in the Southwest during the winter might exacerbate the spread of influenza.
